# The impact of structural embeddedness of neurons on network dynamics

**DOI:** 10.1186/1471-2202-12-S1-P183

**Published:** 2011-07-18

**Authors:** Ioannis Vlachos, Ad Aertsen, Arvind Kumar

**Affiliations:** 1 Bernstein Center Freiburg, University of Freiburg, Germany; 2 Neurobiology & Biophysics, Faculty of Biology, University of Freiburg, Germany

## 

It is a common practice in experimental neuroscience to assess the statistical significance of spiking activity variations, measured from single or multiple neurons. For instance, in behavioral experiments, the probability of an increase in firing rate or correlation strength among a group of neurons is estimated under the assumption that an appropriately chosen null-hypothesis were true. If the probability for observing the experimental results under the null-hypothesis is small, the results are deemed statistically significant and the neural activity is assumed to be functionally related to the task. Thus, it is also implicitly assumed that the statistically significant neural activity must have some effect on the network dynamics. However, this tacit inference is not warranted a priori. That is, the fact that the recorded neuronal activity is not a chance event does not necessarily imply that it will have an impact on local or downstream network activity, particularly when the network is not homogeneously random. Therefore, any strong, or even causal association to behavior is not justified either.

We illustrate this largely ignored point by systematically analyzing the responses of 100 simulated spiking neuron networks, each composed of 10,000 neurons, to external stimulation. All networks had different topologies, however, the average connectivity parameters were kept constant. We measured the population activity and related it to network properties that characterize the way in which the stimulated neurons are embedded in their local environment. To estimate the embeddedness of neurons, we used known metrics from graph theory such as centrality and k-shell decomposition. Our results indicate that the impact neuronal events have on local or downstream network dynamics strongly depends on the *structural embeddedness* of participating neurons. We discuss potential implications of our findings for the analysis of neuronal activity. We also point out additional hurdles that need to be overcome in extracting network function, which go beyond knowledge of the structure and dynamics.

